# Determination of Ascorbic Acid, Total Ascorbic Acid, and Dehydroascorbic Acid in Bee Pollen Using Hydrophilic Interaction Liquid Chromatography-Ultraviolet Detection

**DOI:** 10.3390/molecules25235696

**Published:** 2020-12-03

**Authors:** Meifei Zhu, Jian Tang, Xijuan Tu, Wenbin Chen

**Affiliations:** 1 College of Bee Science, Fujian Agriculture and Forestry University, Fuzhou 350002, China; zmf563563@163.com (M.Z.); t1074643655@163.com (J.T.); 2 College of Animal Science, Fujian Agriculture and Forestry University, Fuzhou 350002, China

**Keywords:** bee pollen, ascorbic acid, total ascorbic acids, dehydroascorbic acid, HILIC

## Abstract

Ascorbic acid (AA) is one of the essential nutrients in bee pollen, however, it is unstable and likely to be oxidized. Generally, the oxidation form (dehydroascorbic acid (DHA)) is considered to have equivalent biological activity as the reduction form. Thus, determination of the total content of AA and DHA would be more accurate for the nutritional analysis of bee pollen. Here we present a simple, sensitive, and reliable method for the determination of AA, total ascorbic acids (TAA), and DHA in rape (*Brassica campestris*), lotus (*Nelumbo nucifera),* and camellia (*Camellia japonica*) bee pollen, which is based on ultrasonic extraction in metaphosphoric acid solution, and analysis using hydrophilic interaction liquid chromatography (HILIC)-ultraviolet detection. Analytical performance of the method was evaluated and validated, then the proposed method was successfully applied in twenty-one bee pollen samples. Results indicated that contents of AA were in the range of 17.54 to 94.01 µg/g, 66.01 to 111.66 µg/g, and 90.04 to 313.02 µg/g for rape, lotus, and camellia bee pollen, respectively. In addition, percentages of DHA in TAA showed good intra-species consistency, with values of 13.7%, 16.5%, and 7.6% in rape, lotus, and camellia bee pollen, respectively. This is the first report on the discriminative determination between AA and DHA in bee pollen matrices. The proposed method would be valuable for the nutritional analysis of bee pollen.

## 1. Introduction

Bee pollen is one of the main sources of food for honeybees. In the process of collecting, honeybees moisten pollen grains with secretions and packs them into the pollen basket. Bee pollen is obtained by pollen trap, which is generally made of a grid and placed on the entrance of hive to remove pollen pellets from honeybee’s legs. As the important nutrient source of honeybee colony, bee pollen is rich in proteins, lipids, sterols, vitamins, and minerals [1,2]. It has been regarded as a natural health food with balanced nutrients, and its potential bioactive and therapeutic properties have made bee pollen a promising therapeutic and nutritional food supplement [3,4,5].

Ascorbic acid (AA) is a water-soluble vitamin, which plays a significant role in human health [6,7]. Since human body does not synthesize AA, it is supplied by exogenous intake. AA can be oxidized to dehydroascorbic acid (DHA) in the condition of high temperature, high pH, light, and in the presence of oxygen [8]. AA has been found to be lost at the food processing and storage stage [9,10]. This loss may be resulted from the oxidation of AA to DHA, and the further degradation to diketogulonic acid [11]. As the processing of bee pollen normally includes a hot-air-drying procedure [12], AA in bee pollen is potentially oxidized to DHA. This oxidation reaction is reversible and DHA can be reduced back to AA in the presence of suitable reductants such as mercapto [13,14] and phosphorus compounds [10,15,16,17]. Studies have shown that the membrane transport of DHA is more efficient than AA, and after crossing membranes DHA can be rapidly reduced to AA by reductase [18]. Thus, the biological activity of DHA has been considered to be equivalent to that of AA, and the determination of total ascorbic acids (TAA) which defined as the sum of both AA and DHA contents would be more accurate for the evaluation of nutritional quality in food analysis [19].

HPLC, which is a powerful technique for the selective and sensitive quantification of natural compounds in complex matrices [20], is the preferred analysis method for determination of AA and DHA [19]. Based on the reversible reaction between AA and DHA, HPLC methods for the analysis of TAA can be divided into two groups. The first one is the oxidation method, in which AA is oxidized to DHA before HPLC separation [21]. Because DHA has little absorbance above 220 nm, the most used ultraviolet (UV) detection technique cannot be utilized directly. Furthermore, DHA is unstable and irreversible hydrolyzed to diketogulonic acid [8,11], thus the oxidized condition must be estimated carefully. Another possibility is the reduction method, in which DHA is reduced to AA by reductants [10,13,15,16]. Since AA is stable in the presence of excessive reductants and shows UV absorption, the reduction method is commonly used [19].

AA content in bee pollen was reported by using 2,6-dichlorophenol-indophenol (DCIP) assays [22,23]. In this assay, the blue dye DCIP is reduced to colorless product by AA, then the content of AA is measured by titrimetric or colorimetric methods. However, this reaction lacks specificity, and many substances in the matrix which are capable of reducing the dye may cause serious interferences [24]. In this paper, we reported the specific determination of AA, TAA, and DHA in three major commercial bee pollen in China, rape (*Brassica campestris*), lotus (*Nelumbo nucifera*), and camellia (*Camellia japonica*), using the hydrophilic interaction liquid chromatography-ultraviolet (HILIC-UV) method to provide high specificity and sensitivity for the analysis of AA. To the best of our knowledge, this is the first report on discriminative determination of AA and DHA in bee pollen by using the proposed methodology.

## 2. Results and Discussion

**Figure 1:**
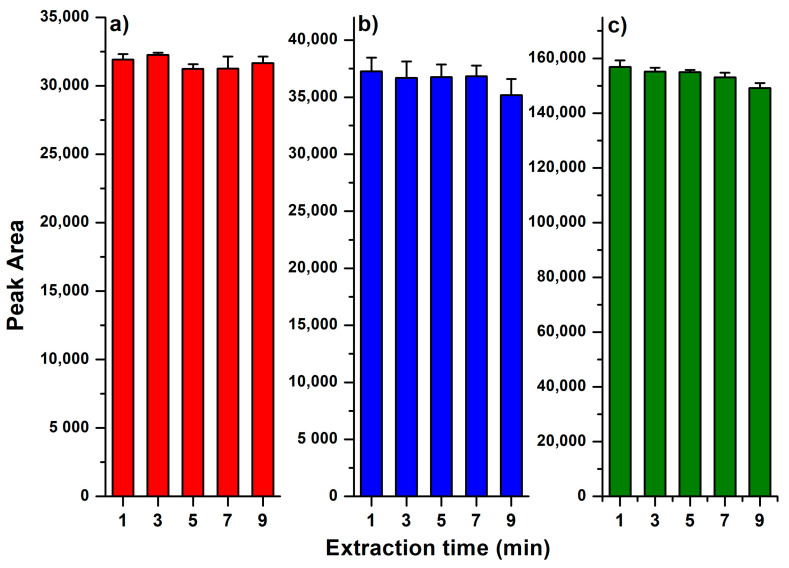
Effect of ultrasonic extraction time on extracted ascorbic acid from different bee pollen samples, (**a**) rape (*Brassica campestris*), (**b**) lotus (*Nelumbo nucifera*), and (**c**) camellia (*Camellia japonica*). The extract under different extraction times were analyzed by hydrophilic interaction liquid chromatography-ultraviolet (HILIC-UV), and the peak area of ascorbic acid (AA) were presented.

The AA in bee pollen samples were extracted by metaphosphoric acid (MPA) solution under ultrasonication. It has been demonstrated that MPA is beneficial for the inhibition of AA oxidation [25]. This acidic condition could prevent the formation of ascorbate, and provide a better recovery of AA. Effect of ultrasonic extraction time on the extracted AA is shown in Figure 1. Results indicated that for all three species of bee pollen, one minute of ultrasonication was sufficient for the extraction of AA into MPA solution. This fast extraction may be attributed to the excellent solubility of AA in MPA solution, and consequently makes the sample preparation very simple and rapid.

**Figure 2:**
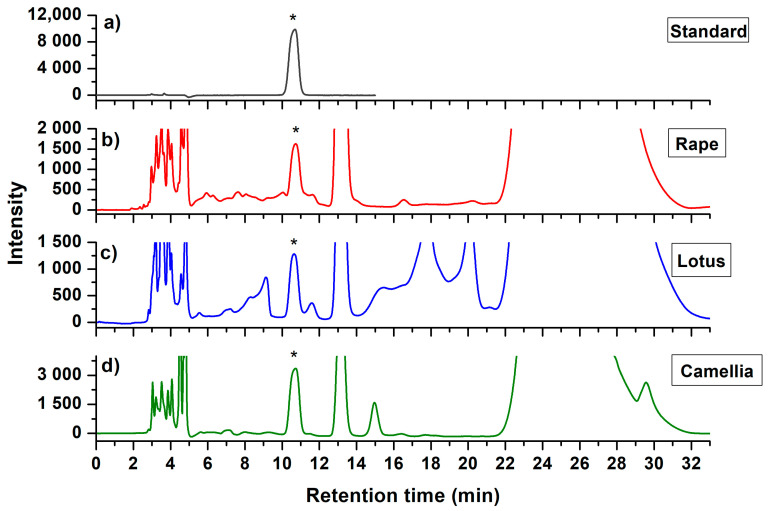
Representative chromatograms of (**a**) ascorbic acid standard, and the final extract of (**b**) rape (*Brassica campestris*), (**c**) lotus (*Nelumbo nucifera*), and (**d**) camellia (*Camellia japonica*) bee pollen. Asterisks indicate the peak of ascorbic acid.

Following the extraction procedure, extract was separated in HILIC with UV detector for the analysis of AA. The column with amide-bonded silica as the stationary phase was used for the HILIC separation due to the compatible with high concentration of MPA solution [26]. The mobile phase consisted of acetonitrile (ACN) and 0.1% formic acid aqueous solution was examined to obtain a general condition for separation of AA in all the three species of bee pollen. Different ratios (*v*/*v*) of ACN ranged from 88% to 94% with interval of 2% were investigated. Under concentration of 88%, the AA peak could not be isolated from interferences in all the three matrices. With the increase of ACN concentration, resolution of AA peak was improved. For rape bee pollen, ACN concentrations with 90% and 92% were both suitable for the separation. While for lotus and camellia bee pollen, only larger than 92% was acceptable for the separation. Meanwhile, as the ACN concentration further increased to 94%, the retention time and peak width of AA were significantly increased. In addition, the peak symmetry was found to be decreased. Therefore, mobile phase composed of 92% ACN was finally proposed for the HILIC separation. Figure 2 represented the chromatograms of AA standard and bee pollen samples under the optimized mobile phase condition with flow rate at 0.8 mL/min. As could be seen in Figure 2a, chromatogram of AA standard showed a well-resolved peak with a retention time (RT) at 10.68 min. Chromatograms of extract from rape (Figure 2b), lotus (Figure 2c), and camellia bee pollen (Figure 2d) all showed the clearly defined AA peaks.

Quantification of AA was performed by external calibration. Good linearity (R^2^ > 0.999) was achieved in the range from 0.2 to 16 µg/mL. According to the literature [27], the instrumental limits of detection (LOD) and quantification (LOQ) were estimated to be 0.06 µg/mL (S/N = 3) and 0.2 µg/mL (S/N = 10) in MPA solution, respectively. Then the LOD and LOQ in bee pollen were calculated by the reported method [15]. Considering that 0.2 g of bee pollen were diluted in 10 mL, this LOD corresponded to a concentration of 3 µg/g bee pollen, and the LOQ corresponded to 10 µg/g bee pollen. Recovery was tested at two fortification levels. Mean recovery percentages were in the range of 90.07~94.82%, 96.98~104.91%, and 90.45~95.41% for rape, lotus, and camellia bee pollen, respectively (Table 1). The coefficient variation of the repeatability (intra-day precision) was 2.25~4.88%, 1.54~4.84%, and 1.69~4.49% for rape, lotus, and camellia bee pollen, respectively. The intermediate precision (inter-day precision) was ranged from 1.85% to 3.87% in these three species of bee pollen. All recovery and precision values were in the acceptable range [28].

**Figure 3:**
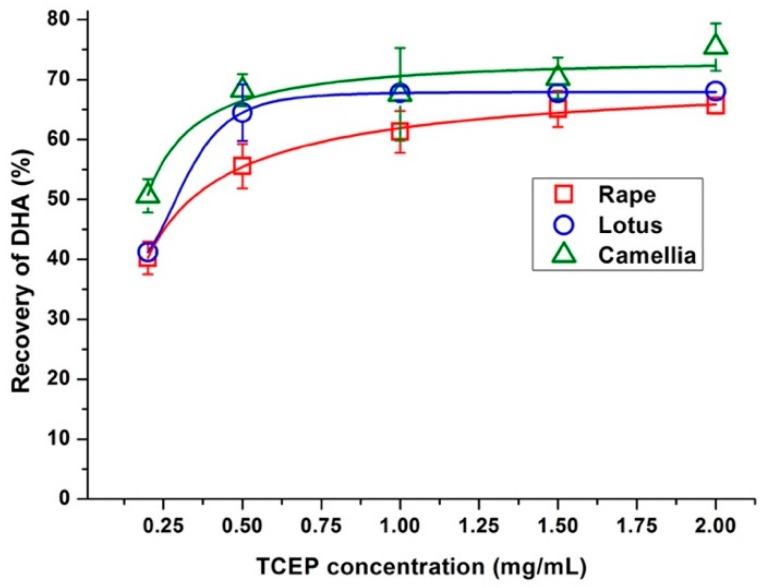
The trends of dehydroascorbic acid (DHA) recovery with increasing the concentration of tris-[2-carboxyethyl] phosphine (TCEP) in rape (*Brassica campestris*), lotus (*Nelumbo nucifera*), and camellia (*Camellia japonica*) bee pollen. The spiked content of DHA in bee pollen samples were 50 µg/g, and the concentration of TCEP in the 10 mL final extract were presented.

For the determination of TAA, reducing reagent was introduced to transform DHA into AA. The reported reagents typically include mercapto based compounds [13,14] and phosphorus-based tris-[2-carboxyethyl] phosphine (TCEP) [10,15,16,17]. Mercapto compounds are efficient only at mildly acidic and neutral conditions, while TCEP shows high reduction efficiency in the MPA solution [29]. Thus, TCEP was selected in the present work. Wechtersbach et al. reported that in MPA solution, DHA was fully reduced to AA by TCEP in less than 20 min [29]. To confirm the conversion of DHA into AA in bee pollen matrices, the recovery of DHA reduced to AA was studied. Bee pollen samples were fortified with DHA at the level of 50 µg/g, and the obtained recoveries under different concentration of TCEP were shown in Figure 3. Results indicated that the recoveries were increased as the concentration of TCEP varied from 0.2 to 1.0 mg/mL, then reached a plateau when the concentration was larger than 1.5 mg/mL. Eventually, TCEP with concentration of 2.0 mg/mL was applied to ensure the accomplishment of DHA reduction. It was important to notice that under this concentration, the calculated recovery values were from 65.6% to 75.4%, which was lower than the recovery of spiked AA as illustrated in Table 1. This lower calculated recovery value may be resulted from the lower purity of commercially available DHA, which was reported to be 80% [29]. After correcting the purity, the accurate recovery should be between 82.0% and 95.4%. These above results indicated that the DHA could be efficiently reduced to AA by the proposed reduction protocol and the present sample preparation method could be applicable for the determination of AA and DHA in these three bee pollen matrices.

**Figure 4:**
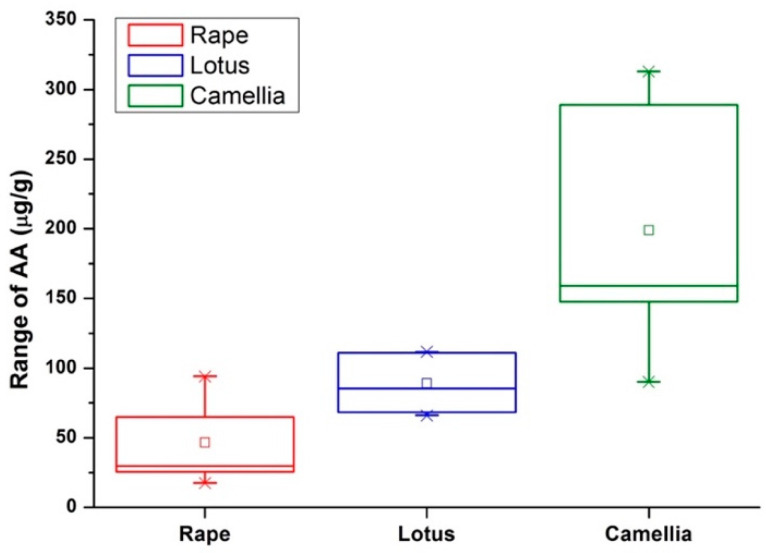
Distribution of the contents of AA in the investigated bee pollen samples.

**Figure 5:**
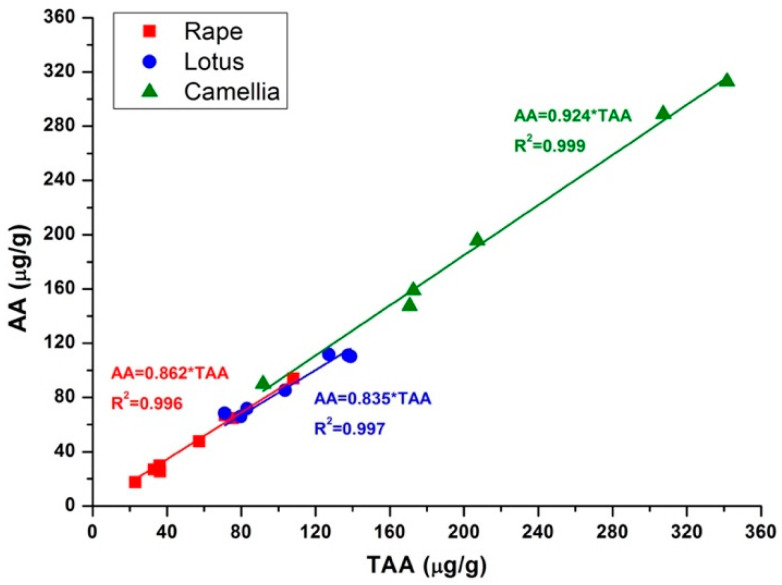
Plots of AA vs. TAA in the investigated bee pollen samples. Linear equation and correlation coefficient (*R*^2^) were presented.

Twenty-one bee pollen samples were analyzed using the proposed method. Results of the detected AA, TAA, and DHA contents in each sample were summarized in Table 2. The distribution of AA contents in different species of bee pollen were compared in Figure 4. It could be observed that the mean AA content was increased as the order of rape (46.63 µg/g) < lotus (89.19 µg/g) < camellia (199.06 µg/g). Additionally, AA contents were distributed with intra-species differences. For instance, camellia bee pollen showed the largest distribution range between 90.04 µg/g and 313.02 µg/g, rape bee pollen was in the range from 17.54 µg/g to 94.01 µg/g, while lotus bee pollen exhibited a relative concentrated range from 66.01 µg/g to 111.66 µg/g. These intra-species differences might be caused by environment conditions such as temperature, light, and storage time, which have significant effects on the oxidization of AA and the degradation of DHA. Furthermore, it was interesting that although the contents of AA were distributed in a wide range, the percentages of AA and DHA in the TAA showed good intra-species consistency. As shown in Figure 5, when the contents of AA were plotted with the contents of TAA, good linearity with the R^2^ value larger than 0.996 were achieved in all the three species of bee pollen. According to the fitting results, percentages of AA in TAA were 86.3%, 83.5%, and 92.4% in rape, lotus, and camellia bee pollen, respectively. This meant that the percentages of DHA in TAA would be about 13.7%, 16.5%, and 7.6% in rape, lotus, and camellia bee pollen, respectively. This stable value of DHA percentage suggests that the irreversible degradation of DHA could be happened in bee pollen samples, and the oxidation of AA into DHA and the further degradation of DHA might be in different equilibrium in these three species of bee pollen.

## 3. Materials and Methods 

### 3.1. Chemicals

HPLC grade acetonitrile and methanol were obtained from Merck (Darmstadt, Germany). Formic acid and metaphosphoric acid were obtained from Sinopharm Chemical Reagent Co., Ltd. (Shanghai, China), AA analytical standard and DHA were purchased from Sigma-Aldrich (Shanghai, China), and TCEP was purchased from Macklin Biochemical Co., Ltd. (Shanghai, China). Commercial bee pollen samples were collected from local markets and stored at −18 °C. Stock solution of AA standard was weekly prepared in MPA solution (5%, *w*/*v*) at the concentration of 0.1 mg/mL. Working solutions of AA standard were prepared daily by further dilution with the MPA solution. TCEP was also prepared in MPA solution at the concentration of 10 mg/mL. DHA was dissolved in methanol with concentration of 0.1 mg/mL. All these solutions were stored at 4 °C until used. Ultrapure water (18.2 MΩ) was used throughout the experiments.

### 3.2. Sample Preparation

Bee pollen samples were homogenized in pulverizer (LINDA-DFY300, Wenling, China) to break up the pollen pellet into powder. For the determination of AA, 0.2 g of homogenized bee pollen powder were placed in a 15 mL tube, then 8 mL of MPA (5%) was added, and the mixture was extracted in an ultrasonic bath (40 Hz, KQ2200B, Kunshan Ultrasonic Instruments Co., Ltd., Kunshan, China) for 1 min. Then the solution was transferred to a 10 mL amber volumetric flask and made up to 10 mL with the MPA solution. Aliquot of the final solution were centrifuged at 10,000 rpm for 10 min, and the supernatant was filtered through a 0.22 μm syringe filters prior to HPLC analysis.

For the determination of TAA, 0.2 g of homogenized bee pollen powder were placed in a 15 mL tube, 2.0 mL of TCEP (10 mg/mL prepared in the MPA solution) and additional MPA solution were added to make the final volume 8 mL, and the mixture was extracted in the ultrasonic bath for 1 min. Then the solution was transferred to a 10 mL amber volumetric flask and made up to 10 mL with the MPA solution. This solution was allowing stay for 20 min at room temperature to accomplish the reduction of DHA. After centrifugation at 10,000 rpm for 10 min, the supernatant was filtered through a 0.22 μm syringe filters for HPLC analysis.

### 3.3. Effect of Ultrasonic Extraction Time

Methods were the same as the determination of AA in Section 3.2, with ultrasonic extraction in different times (1, 3, 5, 7, 9 min).

### 3.4. Effect of TCEP Concentration on the Recovery of DHA

Bee pollen samples were fortified with DHA at the level of 50 μg/g, then the determination of TAA were performed as described in Section 3.2 with different volumes of TCEP solutions (0.2, 0.5, 1.0, 1.5, and 2.0 mL). Control experiments were carried out as above without the fortification of DHA. Then the recovery of DHA was calculated as the percentage of the measured spike relative to the amount of spike added to the sample.

### 3.5. Liquid Chromatography

AA was analyzed using Shimadzu chromatographic system with LC-20AT pump, SIL-20A auto-injector, CBM-20A controller, CTO-20AS oven, and SPD-20A detector. Separation of AA was performed in an Inertsil Amide (GL Sciences) column (5 µm, 4.0 mm × 250 mm). Mobile phase consisted of 0.1% formic acid (solvent A) and ACN (solvent B). Chromatographic conditions were as follows: Elution with 92% solvent B for 15 min for the separation of AA, then post run with 92% to 60% for 1 min and maintained at 60% for 5 min to wash out the matrix compounds, and back to 92% for 2 min and maintained at 92% for 10 min. Flow rate was fixed at 0.8 mL/min, column was maintained at 25 °C, the injection volume was 10 µL, and the UV detector was used at 254 nm.

### 3.6. Method Validation

Quantification of AA was based on calibrations of external standards. The linearity on a seven-point calibration curve was checked ranging from 0.2 to 16 μg/mL, by constructing peak area vs. the concentration of AA. The LOD and LOQ were defined as 3 times and 10 times the signal-noise ratio, respectively [27]. They were determined by serial dilution of the standard solution using the described HPLC conditions and calculated by using the reported method [15]. To evaluate the accuracy and precision of the method, recovery experiment was performed by analyzing samples spiked at two concentration levels. Precision was determined as relative standard deviation (RSD) to the mean recovery in repeatability (intra-day precision, *n* = 6) and intermediate precision (inter-day precision, three days, *n* = 18) analysis.

## 4. Conclusions

We demonstrated that the proposed HILIC-UV method could be used for the determination of AA, TAA, and DHA in rape, lotus, and camellia bee pollen. The sample preparation procedure was simple, rapid, and effective by using ultrasonic extraction in MPA solution. In addition, TCEP conferred the highly efficient reduction of DHA into AA in bee pollen matrices. Finally, the proposed method was fully validated and successfully applied in twenty-one bee pollen samples. The present method would be a suitable approach for the analysis of AA in bee pollen and be valuable for the better understand of bee pollen nutrition.

## Figures and Tables

**Table 1:** The recovery and precision for AA at spiked bee pollen samples.

Bee Pollen	AA, µg/g (Mean ± SD, *n* = 6)	Fortified AA Contents, µg/g	Intra-Day Recovery and Precision						Inter-Day Recovery and Precision	
			Day 1		Day 2		Day 3			
			Mean ± SD (%, *n* = 6)	RSD (%, *n* = 6)	Mean ± SD (%, *n* = 6)	RSD (%, *n* = 6)	Mean ± SD (%, *n* = 6)	RSD (%, *n* = 6)	Mean ± SD (%, *n* = 18)	RSD (%, *n* = 18)
Rape (*Brassica campestris*)	46.55 ± 0.66	20	90.38 ± 2.21	2.44	90.21 ± 3.80	4.21	94.82 ± 4.63	4.88	91.80 ± 3.55	3.87
		40	92.43 ± 2.63	2.84	94.14 ± 2.11	2.25	90.07 ± 2.82	3.13	92.21 ± 2.52	2.73
Lotus (*Nelumbo nucifera*)	71.05 ± 0.97	35	104.91 ± 2.86	2.73	96.98 ± 4.69	4.84	101.53 ± 3.20	3.15	101.14 ± 3.58	3.54
		70	98.75 ± 1.52	1.54	98.35 ± 1.97	2.00	97.47 ± 1.96	2.01	98.19 ± 1.82	1.85
Camellia (*Camellia japonica*)	311.22 ± 5.70	160	95.41 ± 4.28	4.49	90.45 ± 2.03	2.25	90.75 ± 2.30	2.53	92.20 ± 2.87	3.11
		320	90.71 ± 1.53	1.69	95.06 ± 2.21	2.32	92.32 ± 2.14	2.31	92.70 ± 1.96	2.11

**Table 2:** The AA, total ascorbic acids (TAA), and DHA contents in the investigated bee pollen samples.

Bee Pollen	Sample ID	AA, µg/g (Mean ± SD, *n* = 3)	TAA, µg/g (Mean ± SD, *n* = 3)	DHA, µg/g
Rape (*Brassica campestris*)	1	64.77 ± 0.35	75.53 ± 3.43	10.76
	2	94.01 ± 0.58	108.10 ± 0.69	14.09
	3	26.90 ± 0.84	32.99 ± 0.49	6.09
	4	17.54 ± 0.66	22.98 ± 0.57	5.44
	5	47.59 ± 0.70	57.41 ± 1.65	9.82
	6	66.99 ± 0.70	71.15 ± 1.24	4.16
	7	25.55 ± 0.32	36.21 ± 0.38	10.66
	8	29.75 ± 1.26	36.11 ± 1.76	6.36
Lotus (*Nelumbo nucifera*)	1	71.69 ± 0.67	83.13 ± 1.50	11.44
	2	85.31 ± 3.03	103.61 ± 0.46	18.30
	3	110.29 ± 1.51	138.90 ± 2.53	28.61
	4	111.02 ± 1.12	137.65 ± 1.22	26.63
	5	68.37 ± 2.75	71.08 ± 1.18	2.71
	6	111.66 ± 0.54	127.26 ± 1.52	15.6
	7	66.01 ± 0.49	79.67 ± 0.42	13.66
Camellia (*Camellia japonica*)	1	289.05 ± 7.30	307.32 ± 7.85	18.27
	2	195.77 ± 3.10	207.15 ± 2.12	11.38
	3	147.53 ± 0.88	170.69 ± 1.74	23.16
	4	90.04 ± 2.89	91.83 ± 0.54	1.79
	5	158.95 ± 3.29	172.71 ± 1.20	13.76
	6	313.02 ± 3.56	341.65 ± 0.71	28.63
